# Disturbed nitric oxide signalling gives rise to congenital bicuspid aortic valve and aortopathy

**DOI:** 10.1242/dmm.044990

**Published:** 2020-09-28

**Authors:** Joshua C. Peterson, Lambertus J. Wisse, Valerie Wirokromo, Tessa van Herwaarden, Anke M. Smits, Adriana C. Gittenberger-de Groot, Marie-José T. H. Goumans, J. Conny VanMunsteren, Monique R. M. Jongbloed, Marco C. DeRuiter

**Affiliations:** 1 Department of Anatomy and Embryology, Leiden University Medical Center, PO Box 9600, 2300 RC Leiden, The Netherlands; 2 Department of Cardiology, Leiden University Medical Center, PO Box 9600, 2300 RC Leiden, The Netherlands; 3 Department of Chemical Cell Biology, Leiden University Medical Center, PO Box 9600, 2300 RC Leiden, The Netherlands

**Keywords:** NOS3, Aortic dissection, Bicuspid aortic valve, Nitric oxide, Development, Congenital heart disease

## Abstract

Patients with a congenital bicuspid aortic valve (BAV), a valve with two instead of three aortic leaflets, have an increased risk of developing thoracic aneurysms and aortic dissection. The mechanisms underlying BAV-associated aortopathy are poorly understood. This study examined BAV-associated aortopathy in *Nos3^−/−^* mice, a model with congenital BAV formation. A combination of histological examination and *in vivo* ultrasound imaging was used to investigate aortic dilation and dissections in *Nos3^−/−^* mice. Moreover, cell lineage analysis and single-cell RNA sequencing were used to observe the molecular anomalies within vascular smooth muscle cells (VSMCs) of *Nos3^−/−^* mice. Spontaneous aortic dissections were found in ascending aortas located at the sinotubular junction in ∼13% of *Nos3^−/−^* mice. Moreover, *Nos3^−/−^* mice were prone to developing aortic dilations in the proximal and distal ascending aorta during early adulthood. Lower volumes of elastic fibres were found within vessel walls of the ascending aortas of *Nos3^−/−^* mice, as well as incomplete coverage of the aortic inner media by neural crest cell (NCC)-derived VSMCs. VSMCs of *Nos3^−/−^* mice showed downregulation of 15 genes, of which seven were associated with aortic aneurysms and dissections in the human population. Elastin mRNA was most markedly downregulated, followed by fibulin-5 expression, both primary components of elastic fibres. This study demonstrates that, in addition to congenital BAV formation, disrupted endothelial-mediated nitric oxide (NO) signalling in *Nos3^−/−^* mice also causes aortic dilation and dissection, as a consequence of inhibited elastic fibre formation in VSMCs within the ascending aorta.

## Abstract (precis)

**Summary:** Nitric oxide defects link bicuspid aortic valve formation and aortopathy through inhibition of elastic fibre formation in vascular smooth muscle cells within the ascending aorta of *Nos3*^−/−^ mice.

## INTRODUCTION

Patients with a congenital bicuspid aortic valve (BAV) often develop subsequent aortopathy later in adulthood. Clinical studies show that patients with a BAV have a threefold increased chance of developing thoracic aortic aneurysms (Cecconi et al., 2006). This aortopathy is considered generally as a haemodynamic result of the disturbed flow caused by the narrowed opening and position of the two leaflets. Various studies demonstrate that first-degree relatives of BAV patients with a normal tricuspid aortic valve are at increased risk of developing aortic complications (Biner et al., 2009), indicating that both BAV and aneurysm formation represent a variable phenotypical expression of a common genetic defect (Loscalzo et al., 2007). Remodelling of the extracellular matrix (ECM) has been linked to aortopathy. Patients with aortic aneurysms typically display medial degeneration as a result of elastic fibre fragmentation (Isselbacher, 2005). Moreover, reduced collagen deposition has also been observed in patients with ascending aortic aneurysms (de Figueiredo Borges et al., 2008).

Aortic vasculature and valve development are closely related and share common embryonic cell populations. Endothelial cells populating the arterial pole of the heart also contribute to the outflow tract cushions from which the aortic valve develops through epithelial–mesenchymal transition. Cardiac neural crest cells (NCCs) and second heart field-derived cell populations both contribute to the interstitial cells of the aortic valves and the medial vascular smooth muscle cells (VSMCs) and adventitial fibroblasts of the aorta. Studies using mice have determined anomalies in embryonic cardiac lineages that result in BAV (Laforest et al., 2011; Peterson et al., 2018), yet much is unknown about how these cell lineages influence ECM composition and contribute to aortic dissections.

In this study, we examined the effects of disrupted nitric oxide (NO) signalling on the thoracic aorta in *Nos3^−/−^* mice, which is a genetic BAV model with a ∼25% penetrance of the phenotype (Lee et al., 2000; Fernandez et al., 2009; Peterson et al., 2018), to identify developmental processes involved in BAV-associated aortopathy. Understanding early aortic vessel formation is crucial to comprehend the risks involved in aortopathy seen in BAV patients and their tricuspid aortic valve (TAV) relatives.

## RESULTS

### Observation of aortic dissection in *Nos3*^−/−^mice

**Fig. 1.**
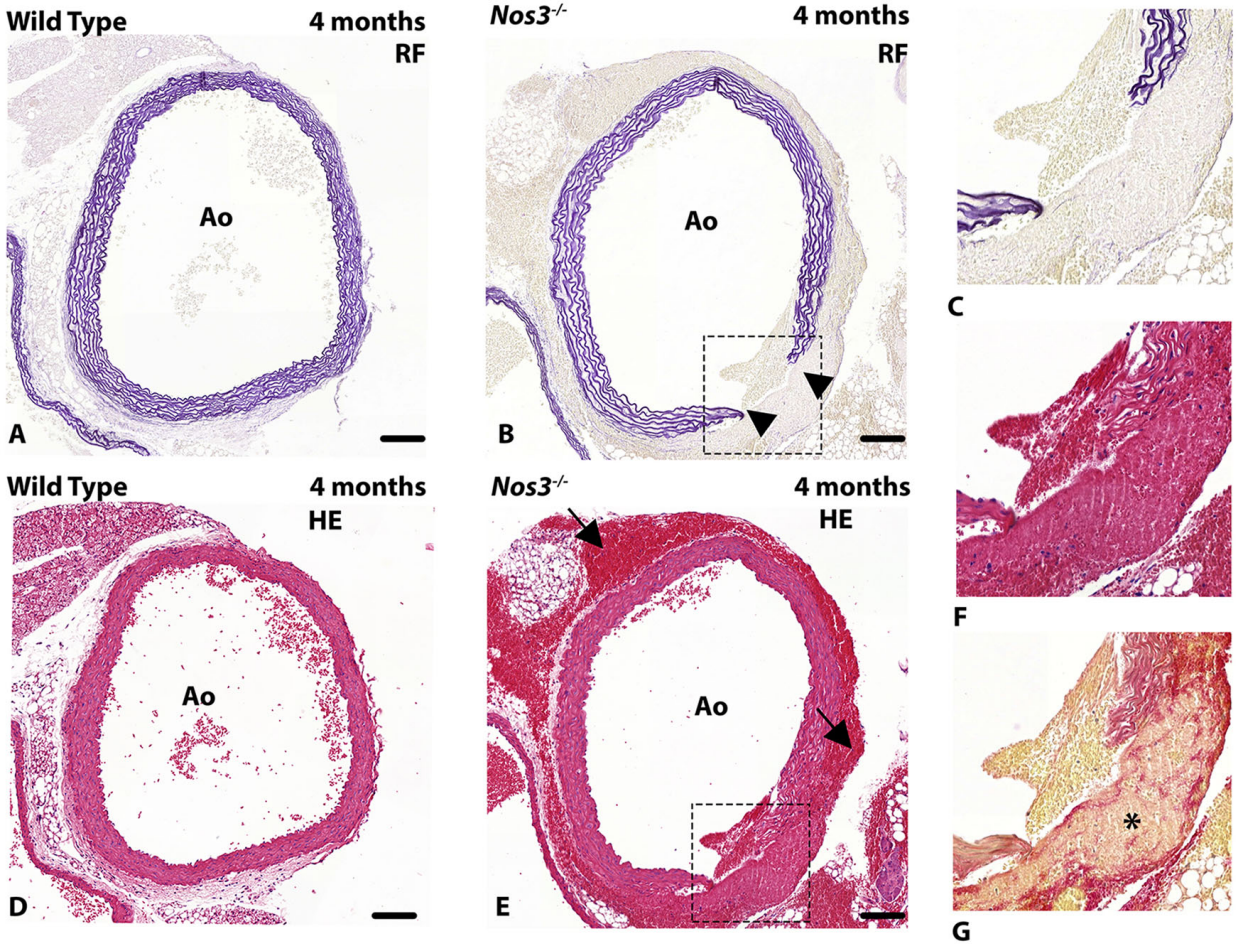
**Aortic dissection in *Nos3*^−/−^ mice.** (A-F) Transverse histological section of the ascending aorta in (A,D) wild-type and (B,C,E,F) *Nos3^−/−^* mice stained with Resorcin-Fuchsin (RF) (A-C) and Haematoxylin-Eosin (HE) (D-F). The *Nos3^−/−^* mice samples reveal rupture (arrowheads) of the elastic lamellae in the ascending aortic vessel wall located at, or slightly above, the sinotubular junction. (G) Adjacent section stained for a combination of collagen (red) and elastin (pink). Tissue remodelling of the adventitia can be observed in aortic vessel walls of dissected *Nos3^−/−^* mice (asterisk). Blood deposits are present in the adventitia and subepicardial space (black arrows). Ao: Aorta. Dashed boxes in B and E indicate regions shown enlarged in C and F, respectively. Scale bars: 100 µm.

The aortic vessel wall is primarily composed of radial sheets of elastin in between layers of VSMCs in the tunica media (Fig. 1). Extracellular collagen contributes mostly to the fibrous structures found in the adventitia of the aortic vessel wall (Fig. S1). Histological examination of aortic vessel walls in *Nos3^−/−^* mice revealed morphological signatures of spontaneous aortic dissections as a result of local disruptions within the aortic vessel wall (Fig. 1; Fig. S1). The aortic dissection was located slightly above the sinotubular junction in *Nos3^−/−^* mice. Adventitial tissue remodelling, low in elastin and collagen content, in response to aortic dissection was observed in the dissected aortic vessel wall of *Nos3^−/−^* mice (Fig. 1G; Fig. S1).

The spontaneous development of aortic dissections seen in *Nos3^−/−^* mice were sparsely distributed within the dataset, occurring in ∼13% of *Nos3^−/−^* mice (4/31 *Nos3^−/−^* mice) ranging in stages from 1 month to 11 months of age. Dissection occurred in both BAV (*N*=1) (Fig. S1) and TAV (*N*=3) *Nos3^−/−^* mice. Survival analysis indicated no difference in the temporal distribution of spontaneous death events between wild-type and *Nos3^−/−^* populations (Fig. S2).

### *Nos3*^−/−^ mice develop aortic dilation early in adulthood

**Fig. 2.**
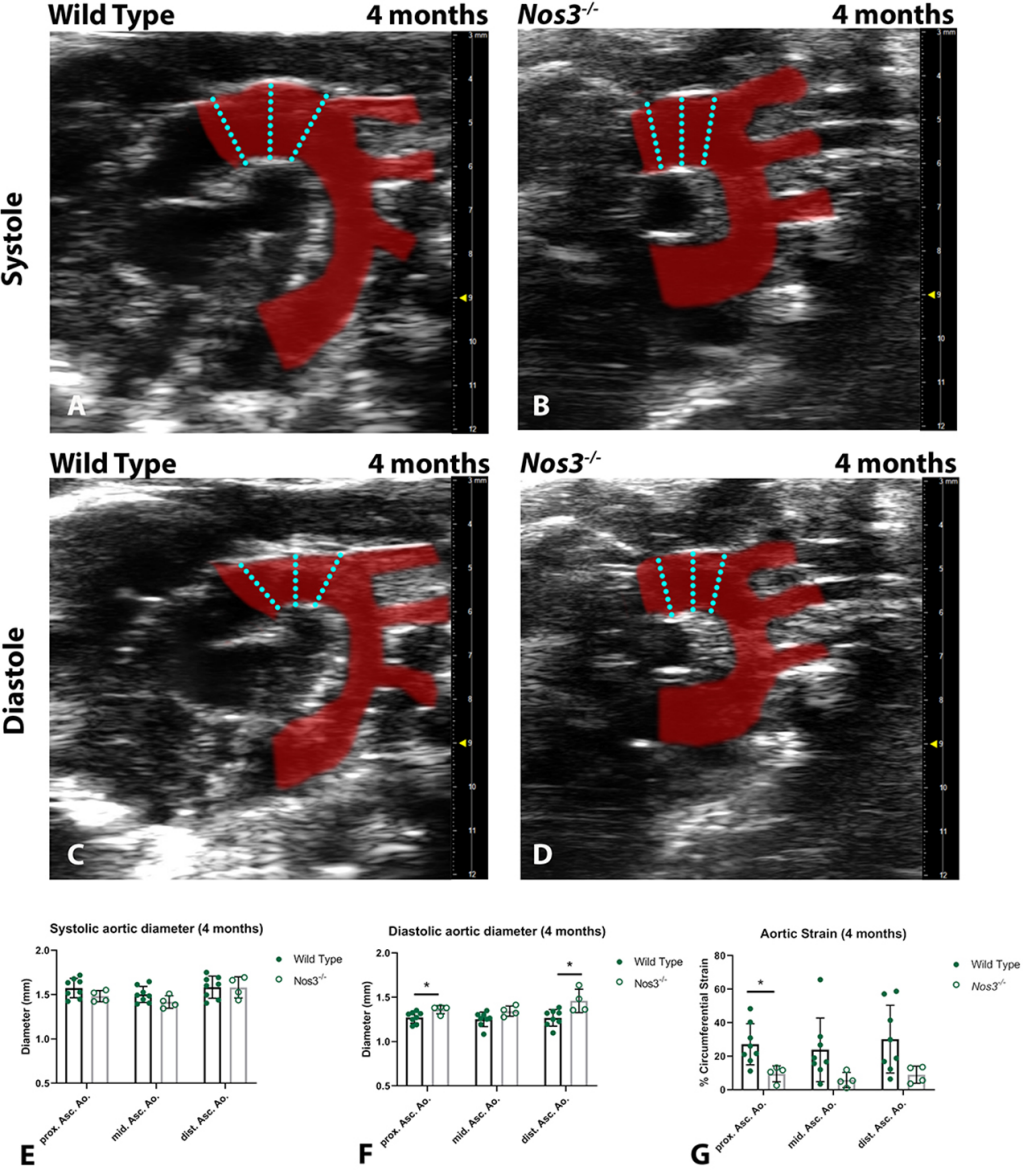
***Nos3^−/−^* mice develop aortic dilation at 4 months.** (A,B) Ultrasound images of the aorta (red) during systole in (A) wild-type and (B) *Nos3^−/−^* mice, displaying the peak systolic diameter of the aorta. (C,D) During diastole, aortic diameter reaches maximum constriction in (C) wild-type and (D) *Nos3^−/−^* mice. (E,F) Aortic diameter was measured in 4-month-old wild-type (*N*=8) and *Nos3^−/−^* (*N*=4) mice at the proximal, mid- and distal ascending aorta (prox. Asc. Ao., mid. Asc. Ao., dist. Asc. Ao., respectively). Cyan dotted lines indicate measurement locations of proximal (left), mid and distal (right) locations of the ascending aorta. (E) Peak systole and (F) peak diastole diameters. *Nos3^−/−^* mice have significantly larger diastolic diameters than wild-type mice. (G) Circumferential Green–Lagrange strain of the aorta was found to be significantly lower in the ascending aorta of *Nos3^−/−^* mice. Data in E-G are mean±s.d. *P<0.05 (two-tailed Student's *t*-test).

To examine whether the aortic dissections coincide with an increased aortic diameter, ultrasound imaging was used to visualize the aorta of wild-type and *Nos3^−/−^* mice *in vivo* (Fig. 2A-D). Careful measurements of the proximal, mid-, and distal ascending aorta were made to determine aortic diameters during systole and diastole (Fig. 2E,F). Aortic ultrasound measurements in 4-month-old adult mice showed no difference in aortic diameter during peak systole compared to that of wild-type mice (Fig. 2E). However, peak diastolic aortic measurements determined significantly larger aortic diameters in the proximal and distal ascending aorta of *Nos3^−/−^* mice compared to those of wild-type mice (Fig. 2F). Moreover, calculations of aortic strain determined significant reductions in circumferential strain in the proximal ascending aorta of *Nos3^−/−^* mice (Fig. 2G).

### Reduced elastic fibres in ascending aortic vessel walls of *Nos3*^−/−^mice

**Fig. 3.**
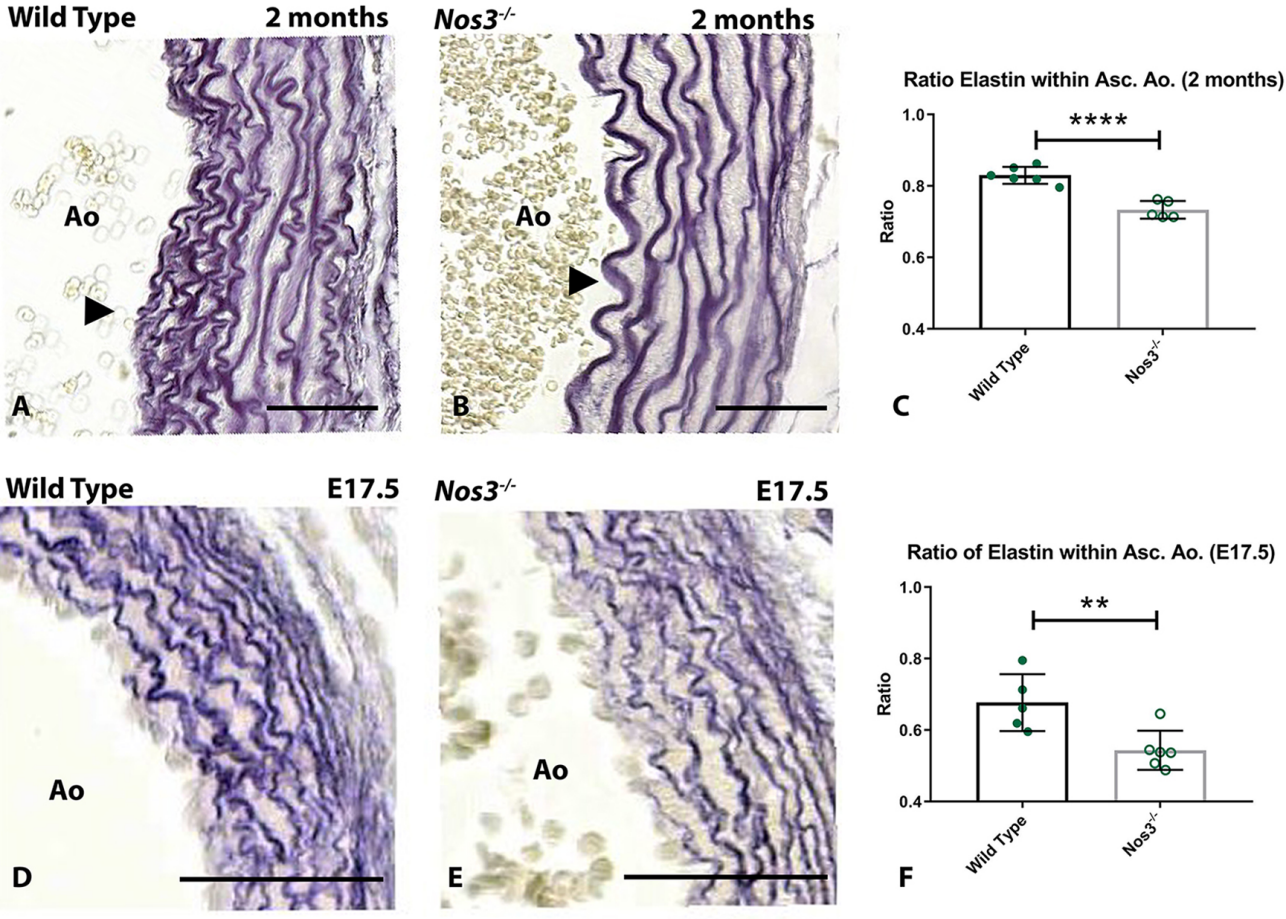
***Nos3^−/−^* aortic walls contain less elastin than those of wild-type mice.** (A,B) Transverse sections of aortic walls of adult (A) wild-type and (B) *Nos3^−/−^* mice stained with Resorcin-Fuchsin to visualize elastin deposited as elastic fibres within the ascending aortic (Asc. Ao.) vessel wall. (C) Volumetric quantification of elastin within the ascending aortic vessel wall of wild-type (*N*=6) and *Nos3^−/−^* (*N*=5) adult (2 months) mice shows a reduction of elastin within aortic vessels of *Nos3^−/−^* mice. (D,E) Resorcin-Fuchsin staining of ascending aorta vessel walls of embryonic (E17.5) (D) wild-type and (E) *Nos3^−/−^* mice. (F) Volumetric quantification of elastic fibres in wild-type (*N*=5) and *Nos3^−/−^* (*N*=6) embryos also shows significant reductions in volume of elastin within the ascending aorta in *Nos3^−/−^* embryos, indicating impaired elastin production during embryogenesis. Morphological smooth elastin fibres, instead of densely packed sinuous lamellae are observed within the inner media (arrowheads) of the aortic wall in *Nos3^−/−^* adult mice. Ao: Aorta. Data in C and F are mean±s.d. ****P<0.0001, **P<0.01 (two-tailed Student’s *t*-test). Scale bars: 50 µm.

The morphologic structure of the ECM within the ascending aorta was analysed at adult as well as embryonic (E17.5) stages of development to examine the onset of vascular wall pathology (Fig. 3). Volumetric quantification of elastic lamellae within the vessel wall of the ascending aorta showed significant reductions in the volume of elastic fibres within vessel walls of *Nos3^−/−^* mice at embryonic as well as adult stages. Morphological comparison of the elastic lamellae in aortic vessel walls indicated that disruptions in elastic fibres impacted the inner medial region of the aortic vessel wall. The aortic vessel wall of wild-type mice consisted of an inner media of densely packed sinuous elastic lamellae and an outer media of smoothly aligned elastin lamellae (Fig. 3A). In contrast, the aortic vessel wall of adult *Nos3^−/−^* mice solely developed smoothly aligned elastin lamellae throughout the complete aortic vessel wall (Fig. 3B).

In addition to elastin, collagen deposition was also examined in aortic vessel walls of adult and embryonic mice (Fig. S3). Volumetric collagen analysis of medial and adventitial aortic collagen deposition determined no significant difference between wild-type and *Nos3^−/−^* mice, indicating that the *Nos3* mutation does not impact collagen deposition within ascending aortic walls of embryonic or adult mice but specifically affects formation of elastic lamellae in the aortic vessel wall (Fig. S3E-H).

### NCC populations are reduced in aortic vessel walls of *Nos3*^−/−^ embryos

**Fig. 4.**
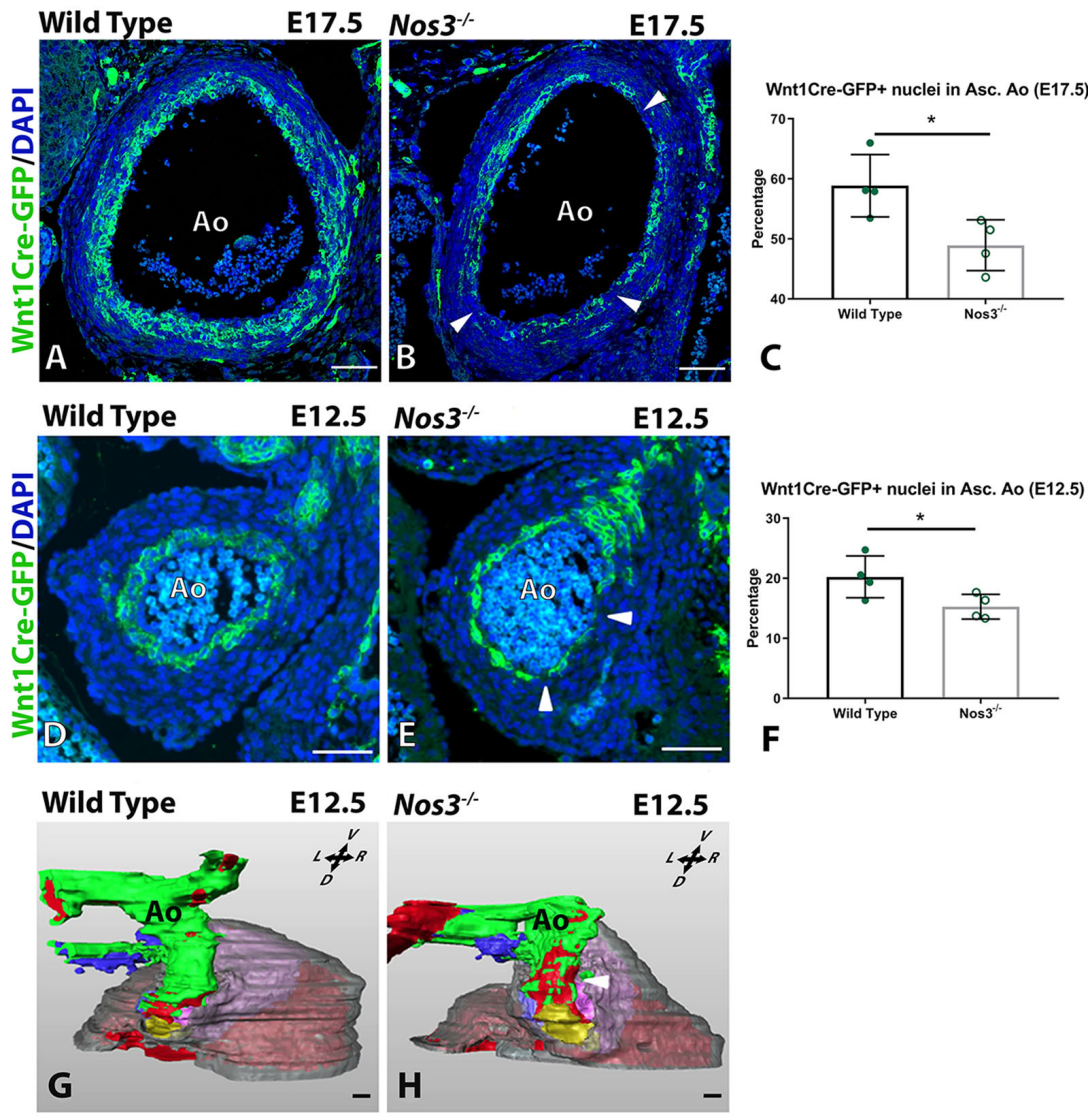
**Reduction of the NCC population in aortic vessel walls of *Nos3*^−/−^ embryos.** (A,B) Immunofluorescence staining of NCC-derived cells (Wnt1Cre-GFP, green) in transverse sections of the ascending aortic vessel walls of (A) *Wnt1Cre^+^;mTmG* and (B) *Nos3^−/−^;Wnt1Cre^+^;mTmG* embryos. (C) Lineage quantification of NCC-derived cells showed a reduced contribution of NCC-derived cells in the vessel wall of the ascending aorta (Asc. Ao.) in *Nos3^−/−^* (*N*=4) embryos when compared to wild-type embryos (*N*=4) at E17.5. (D,E) Immunofluorescence staining of NCC-derived cells (Wnt1Cre-GFP, green) in the ascending aortic vessel wall of E12.5 (D) wild-type and (E) *Nos3^−/−^* embryos. (F) Cell lineage analysis at E12.5 also showed reduced contribution of NCC-derived cells within the ascending aorta of *Nos3^−/−^* (*N*=4) embryos when compared to age-matched wild-type embryos (*N*=4). Incomplete coverage of the inner media by NCC-derived cells was observed in the inner media in *Nos3^−/−^* of both E17.5 and E12.5 embryos (white arrowheads). Nuclear DAPI staining is shown in blue. (G,H) 3D reconstruction of E12.5 wild-type *Wnt1Cre^+^* hearts (G) showed NCC-derived cells (green) surrounding the complete lumen (red) of the ascending aorta. In contrast to the wild type, E12.5 *Nos3*^−/−^; *Wnt1Cre^+^*;*mTmG* embryos (H) showed a reduced number of NCCs throughout the ascending aorta as well as an incomplete NCC coverage of the aortic root and proximal ascending aorta. White arrowhead indicates incomplete coverage of the inner media by NCC-derived cells. Colour coding: myocardium, transparent grey; parietal outflow tract cushion, purple; non-coronary leaflet, yellow; septal outflow tract cushion, light blue; pulmonary trunk, dark blue. Ao, aorta; L, left; R, right; D, dorsal; V, ventral. Data in C and F are mean±s.d. *P<0.05 (two-tailed Student's *t*-test). Scale bars: 50 µm.

The observation of elastin disruption within the inner media suggested a possible role of the NCC lineage in aortopathy. Previous findings from our lab already established NCC lineage disruption in aortic valves during cushion development in *Nos3^−/−^* embryos (Peterson et al., 2018). Genetic lineage tracing using *Wnt1Cre;mTmG* and *Nos3^−/−^;Wnt1Cre;mTmG* embryos (E17.5 and E12.5) showed that NCC-derived VSMCs line the entire inner media of the ascending aorta (Fig. 4; Fig. S4). Comparison of the NCC-derived cell populations in the ascending aortic vessel wall between wild-type and *Nos3^−/−^* embryos showed a significant reduction of NCCs in the aortic vessel wall of *Nos3^−/−^* embryos at both E17.5 and E12.5 (Fig. 4C,F). Close morphological examination revealed incomplete coverage in the inner media of the ascending aorta by NCC-derived cells in *Nos3^−/−^* embryos. Three-dimensional reconstruction of the E12.5 outflow tract indicated that the reduction in NCC-derived cells in *Nos3^−/−^* embryos is limited to the ascending aorta (Fig. 4G,H).

### Single-cell RNA sequencing reveals downregulation of genes associated with aortopathy in VSMCs of *Nos3*^−/−^ mice

**Fig. 5.**
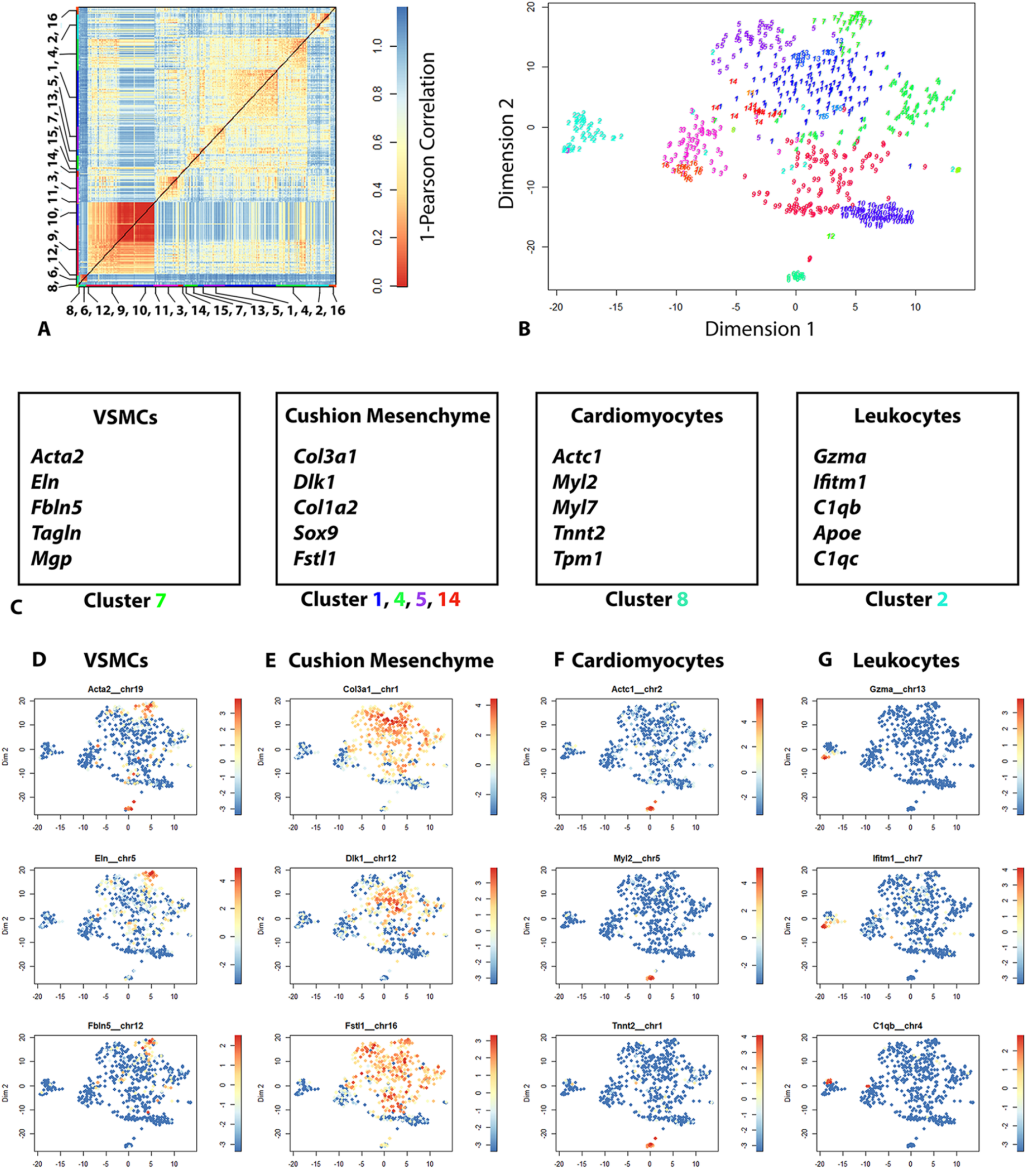
**Clustering of cardiac outflow tract cells based on gene expression.** (A) Heatmap depicting Pearson correlation distance in cell-to-cell transcriptomes of 607 cells obtained from wild-type outflow tract tissue at E12.5. K-medoids clustering identified 16 clusters. Cluster numbers are indicated on the heatmap. (B) t-SNE map showing cell clusters based on affinity in RNA transcriptome profiles corresponding to different cell types. (C) Tables of established marker genes used to identify cell types corresponding to cluster numbers indicated in B. (D-G) t-SNE maps showing relative RNA expression of cell specific markers indicating (D) vascular smooth muscle cells (VSMCs), (E) cushion mesenchyme, (F) cardiomyocytes and (G) leukocytes. Data are shown as normalized transcript counts on a colour-coded logarithmic scale (log2).

To investigate the effects of the *Nos3* mutation on a transcriptional level, single-cell RNA-seq was used on the murine outflow tract of E12.5 embryos. K-medoids clustering using the Race-ID3 algorithm (Herman et al., 2018) defined 16 cell clusters based on similarities in cellular gene expression in the outflow tract of wild-type origin (Fig. 5). Individual clusters were examined for known marker genes to identify cell types corresponding to each cluster. Using this approach we identified multiple cell types, namely VSMCs, cushion mesenchyme, cardiomyocytes and leukocytes.

**Fig. 6.**
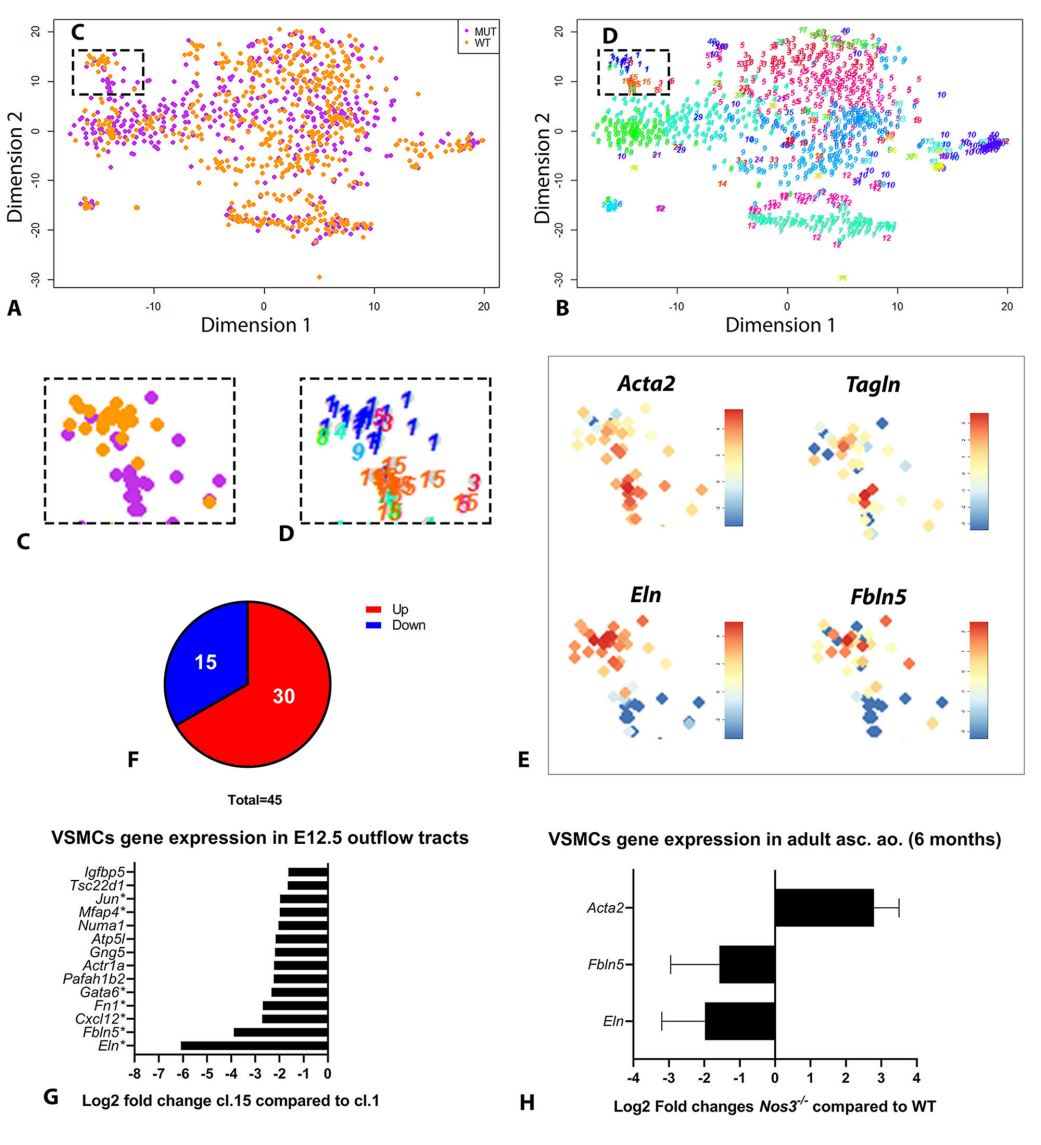
**Single-cell transcriptome analysis of wild-type and Nos3^−/−^ mice.** (A,B) t-SNE map indicating transcriptome similarities among 1099 single cells. (A) Colours highlight the genotype of cells (wild type, orange; *Nos3^−/−^*, purple). Dashed box indicates the VSMC cluster shown in C. (B) Numbers highlight the cluster numbers identified. Dashed box indicates the VSMC cluster shown in D. (C,D) Enlargement of the t-SNE map from A and B, focusing on the VSMC cluster, showing segregation of wild-type and *Nos3^−/−^* VSMC cells. (E) t-SNE maps showing relative expression of the indicated RNAs in the VSMC clusters. Data are shown as normalized transcript counts on a colour-coded logarithmic scale (log2). (F) Pie chart showing the number of significantly (*P*<0.05) up- and down-regulated genes in *Nos3^−/−^* VSMCs (cluster 15) compared to wild-type VSMCs (cluster 1) at E12.5. (G) Expression of the 15 significantly downregulated genes in the *Nos3^−/−^* VSMC cluster (cluster 15, cl.15) compared to the VSMCs in the wild-type cluster (cluster 1, cl.1). Known genes linked to aneurysm formation have been marked with an asterisk. (H) qPCR of the top three differentially expressed genes found in E12.5 VSCMs by single-cell RNA-seq in adult (6 months) VSMCs from the ascending aorta. *N*=5 for both wild-type (WT) and *Nos3^−/−^* mice. *Rpl32* was used as the reference gene. Data are mean±s.d.

Combined clustering of wild-type and *Nos3^−/−^* single-cell transcriptomes allowed for an unbiased assessment of cell type differences, because clustering depends on cellular similarity based on gene expression. Two clusters were found in close proximity of each other, in which wild-type (cluster 1) and *Nos3^−/−^* (cluster 15)-derived cells formed separate near homogenous groups (Fig. 6A-D). Based on the relative high *Acta2* and *Tagln* RNA expression levels within these clusters, they were identified as VSMCs. Differential gene expression analysis between wild-type and *Nos3^−/−^* VSMC clusters revealed significant differences in gene expression of a total of 45 genes (30 upregulated and 15 downregulated genes) of which the top upregulated gene was *Acta2*, and the top downregulated gene was *Eln* in *Nos3^−/−^* VSMCs. (Fig. 6E,F; Fig. S5). We subsequently focused on downregulated genes, because BAVs and BAV-related aortopathy are often associated with gene mutations resulting in downregulated gene expression (Prakash et al., 2014). The absence of *Nos3*-induced NO signalling resulted most notably in the downregulation of *Eln* transcription, a gene encoding for elastin – a major component of elastic fibres (Fig. 6E,G). VSMCs of *Nos3^−/−^* mice also had decreased expression of *Fbln5*, which translates to fibulin-5, another important protein that directly interacts with elastin for the formation of elastic fibres in the ECM (Midwood and Schwarzbauer, 2002; Yanagisawa et al., 2002). Interestingly, multiple genes – *Eln*, *Fbln5*, *Cxcl12*, *Fn1*, *Gata6* and *Mfap4* – were found to be downregulated in *Nos3^−/−^* VSMCs; genes which are all associated with BAV and aneurysm formation (Midwood and Schwarzbauer, 2002; Ogata et al., 2005; Paloschi et al., 2011; Orriols et al., 2016; Girdauskas et al., 2017; Zhang et al., 2018) (Fig. 6G). To ascertain that the changes in gene expression were not limited to stages of embryonic development, qPCR was performed for the top three differentially expressed genes on ascending aortic tissues from adult wild-type and *Nos3^−/−^* mice. qPCR analysis determined similar upregulation of *Acta2* and downregulation of *Eln* and *Fbln5* expression as seen in the RNA-seq analysis of E12.5 embryos, indicating that these expression changes persist into adulthood (Fig. 6H; Fig. S6).

### Genetic misregulation translates into altered protein expression phenotypes and is most pronounced in NCC-derived VSMCs of *Nos3*^−/−^ mice

**Fig. 7.**
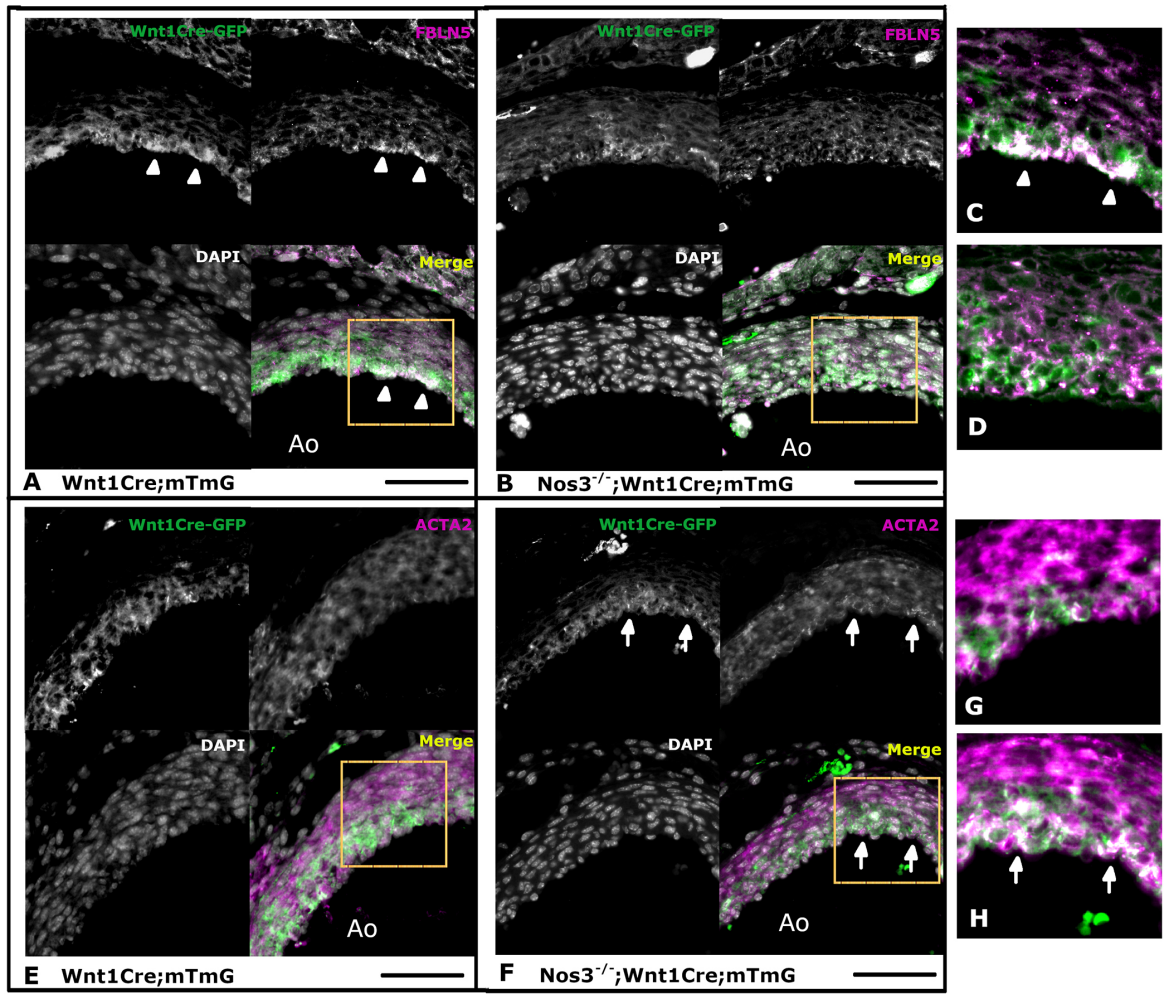
**Genetic variations translate to misregulated protein expression in neural-crest-derived vascular smooth muscle cells of *Nos3*^−/−^embryos.** Fluorescence microscopy images of transversal sections of the aortic vessel wall in *Wnt1Cre^+^;mTmG* and *Nos3^−/−^;Wnt1Cre^+^;mTmG* embryos aged E17.5. (A,C) Neural-crest-derived VSMCs (Wnt1Cre-GFP, green), line the inner media of the aortic vessel wall and express FBLN5 (magenta) (see arrowheads). (B,D) *Nos3*^−/−^ neural-crest-derived VSMCs show reduced accumulation of FBLN5 compared to those in wild-type embryos. Boxes in A and B indicate regions shown enlarged in C and D, respectively. Enlarged images show Wnt1Cre-GFP and FBLN5 channels. (E,F) Neural-crest-derived VSMCs (Wnt1Cre-GFP, green) express ACTA2 (magenta), showing increased expression of ACTA2 in neural-crest-derived VSMCs in *Nos3*^−/−^ embryos (indicated by arrows). Boxes indicate regions shown enlarged in G and H. (G,H) Enlarged views of the Wnt1Cre-GFP and ACTA2 channels of E and F, respectively. Ao, Aorta. Nuclear staining using DAPI is shown in grey. Scale bars: 50 µm.

To examine the effects of the aberrant RNA expression profiles found in the VSMCs of *Nos3^−/−^* embryos, immunofluorescent antibody stainings were performed to examine localized alterations in protein translation within the aortic vessel wall of E17.5 embryos (Fig. 7). Interestingly, FBLN5 expression was most pronounced in NCC-derived VSMCs of wild-type embryos (Fig. 7A,C). Conversely, NCC-derived VSMCs of *Nos3^−/−^* embryos did not accumulate FBLN5, in accordance with the phenotype of reduced *Fbln5* expression (Fig. 7B,D). Moreover, in both wild-type and *Nos3^−/−^* embryos, ACTA2 expression was more pronounced in NCC-derived VSMCs than in VSMCs from a different origin (Fig. 7E-H; Fig. S4). Nonetheless, the differences observed in ACTA2 expression between wild-type and *Nos3^−/−^* embryos support a phenotype of *Acta2* overexpression within the NCC-derived VSMCs of *Nos3^−/−^* embryos.

The protein expression dynamics of both FBLN5 and ACTA2 were in agreement with the findings of those predicted by single-cell RNA-seq and qPCR. These findings demonstrate the importance of NO signalling for maintaining the vessel wall integrity of the ascending aorta.

## DISCUSSION

Patients with a BAV have a higher risk of developing aortopathy of the ascending aorta. The exact mechanisms through which BAV-associated aortopathy arises are still poorly understood. Knowledge of the underlying processes could advance patient risk assessments and aid in the development of early diagnostic tools.

We examined the impact of NO depletion to identify effects of BAV-associated aortopathy in mice. We describe for the first time that *Nos3^−/−^* mice develop dissections in the ascending aorta as a result of effects on signalling pathways involved in elastic fibre formation.

Studies by Koenig and colleagues have reported evidence of aortopathy in mice with haploinsufficiency of *Notch1* in a *Nos3^−/−^* mixed background (Koenig et al., 2015). Later studies by the same group, however, reported that *Notch1* haploinsufficiency in 129SV mice also causes ascending aortic aneurysm, making the role of *Nos3* in aortopathy less clear (Koenig et al., 2017). Reports examining HPH-1 mice, a mouse model with uncoupled NOS3, showed rapidly developing abdominal aortic aneurysms as well as aortic rupture upon infusion of angiotensin II (Gao et al., 2012). Moreover, Fan and colleagues showed that angiotensin II infusion can also lead to abdominal aortic dissection through endothelial-mediated reactive oxygen signalling in wild-type mice (Fan et al., 2014). Reports by Kuhlencordt and colleagues show that double-knockout *Apoe/Nos3* models develop severe cardiovascular complications, including spontaneous abdominal aortic aneurysms and dissection (Kuhlencordt et al., 2001). These studies all suggest that, although *Nos3* is an important gatekeeper of the aortic vessel wall that acts in combination with other factors to maintain aortic stability, single gene loss of *Nos3* function does not result in aortopathy.

The spontaneous aortic dissections as found in *Nos3^−/−^* mice in this study might have been overlooked by previous studies because the age of onset was distributed over a period of 11 months. *Nos3^−/−^* mice in which dissections were found were acquired during routine investigations. Haemorrhages found within dissected mice were limited to the subepicardial space and were not found within the pericardial cavity or the mediastinum. This could suggest that these mice were collected during a window in which the mice were still viable but at high risk to succumb to further aortic deterioration. The mortality rate in humans is known to increase to 70% within 48 h after aortic dissection (Hagan et al., 2000; Criado, 2011) and *Nos3^−/−^* mice might face an equal rapid increase of mortality rate after the onset of aortic dissection. Nevertheless, the observation of aortic remodelling in *Nos3^−/−^* mice might challenge this concept. The survival analysis did not reveal a specific interval at which spontaneous deaths of *Nos3^−/−^* mice differed from those of wild-type populations, complicating the acquisition of mice prone to dissect. Future studies should look more specifically into the mortality rate related to aortic dissection in *Nos3^−/−^* mice to better understand the timing and risks involved in the development of an aortic dissection.

Ultrasound measurements of the aorta showed increased aortic diameters in 4-month-old *Nos3^−/−^* mice. These results suggest that the aortic vessel wall developed structural aortic dilations in adulthood, similar to aortic aneurysm development observed in BAV patients (Cecconi et al., 2006). Circumferential aortic strain, a measure of aortic elasticity, is known to decrease with age and has been explored in clinical studies to examine aortic stiffness; it is considered an important cardiovascular risk factor for patient health (Redheuil et al., 2010). Moreover, in mouse models of Marfan syndrome, circumferential aortic strain has been shown to correlate with elastin fragmentation and reduced elastic lamellae in aortic vessel walls (Mariko et al., 2011; Chen et al., 2019).

The aorta and the aortic valve have a similar developmental origin involving contributions of endothelial, NCC and second heart field lineages. BAV patients have increased risk of developing aneurysms and dissections of the ascending aorta but not of the descending aorta (Biner et al., 2009). NCCs are known to contribute to the formation of the VSMCs of the aortic root, ascending aorta and aortic arch. A reduction in the NCC-derived populations was observed in the ascending aorta of E12.5 and E17.5 *Nos3^−/−^* embryos, and was most notable in the region of the commissures. NCCs have been reported to accumulate in the commissures; however, their function there is still poorly understood (Badger et al., 2010; Orriols et al., 2016). Previous studies have shown the importance of correct NCC distribution for proper aortic valve and outflow tract formation (Paloschi et al., 2011; Peterson et al., 2018; Zhang et al., 2018). Moreover, BAV patients and their first-degree relatives have increased risk of aortopathy, suggesting that the underlying mechanisms are not limited solely to BAV cases (Biner et al., 2009). Our findings were derived from *Nos3^−/−^* mice that were not selected *a priori* for BAV or TAV phenotypes, but *Nos3^−/−^* mice have been known to develop BAV in ∼25% of cases (Fernandez et al., 2009; Peterson et al., 2018). This study found aortic dissection in both TAV and BAV *Nos3^−/−^* mice, similar to patient observations, suggesting a role for NO signalling in aortic development in both humans and mice.

This study found a significant downregulation of both protein and RNA expression of elastin in VSMCs, resulting in aortic dissection in *Nos3^−/−^* mice. Interestingly, clinical studies show that the elastin content is generally decreased in the ascending aortic wall of dissected patients when compared to controls (Cattell et al., 1993). Decreased elastin concentrations within the aortic wall are strongly correlated with decreased expression of *Fbln5* in patients with ascending aortic dissection, similar to our findings using *Nos3^−/−^* mice (Wang et al., 2005). Whereas elastic fibre degeneration in dissected aortic patients is often attributed to increased activity of metalloproteases (MMPs) (Zhang et al., 2011), *Nos3^−/−^* mice have decreased NO production, which results in inhibition of MMP activity (Ridnour et al., 2007). Elastic fibre degeneration might also be the result of reduced *Fbln5* expression, because FBLN5 can function as scaffolding protein during elastic fibre assembly (Midwood and Schwarzbauer, 2002). *Fbln5^−/−^* mice show reduced contractility in the thoracic aorta (Murtada et al., 2016) and develop hypertension (Le et al., 2014) but do not give rise to aortic aneurysms or aortic dissection. Similarly, *Eln^+/−^* mice also show reduced aortic contractility and increased blood pressure (Jiang et al., 2000; Jain et al., 2011). In humans, hypertension is the single most important risk factor for aortic dissection (Hagan et al., 2000). Interestingly *Nos3^−/−^* mice have been described to also exhibit high blood pressure (Huang et al., 1995). The exact mechanisms by which *Nos3* mutation gives rise to aortic dilation and dissection is not yet fully understood. Given that NCC populations were found to be reduced in size within the inner media of *Nos3^−/−^* embryos, this might suggest a novel role for NO signalling during development. Genetic predisposition of aortic root aneurysm pathogenesis has been observed as a result of lineage-specific events related to NCC-derived VSMCs in Loeys-Dietz syndrome, a disease in which BAVs are more frequently observed than in the general population (MacCarrick et al., 2014; MacFarlane et al., 2019). We found that most changes related to gene expression within the aortic vessel wall were most prominent in NCC-derived VSMCs of *Nos3^−/−^* mice. The NCC-derived VSMCs populate the inner media of the aorta and might depend on the paracrine cues of NO signalling to function properly. Interestingly, Kong and colleagues have shown that inhibition of NO signalling during development affects cranial neural crest patterning, differentiation and convergence in the pharyngeal arch, demonstrating a coordinating role of NO signalling during development (Kong et al., 2014). Moreover, Suvorava and colleagues have shown that *Nos3*^−/−^ rescue through additional NO supplementation does not result in reduced hypertension in 3- to 4-month adult *Nos3^−/−^* mice, supporting an extra-endothelial role of *Nos3* (Suvorava et al., 2015). Effects of NO signalling on cellular function are diverse as NO is known to act on multiple kinase signalling cascades (Schindler and Bogdan, 2001) and affect multiple transcription factors through NF-kB, c-Fos–Jun, Sp1, Egr-1, VDR–RXR and HIF-1 interactions (Bogdan, 2001; Hemish et al., 2003). This makes it challenging to interpret primary pathways involved during outflow tract formation. Future studies focusing on the molecular interactions between endothelial cells and NCCs during development should provide more insight into the signalling routes through which *Nos3* acts during development.

The role of *Nos3* in thoracic aneurysms, aortic dissections and BAV in humans is still poorly understood. In multiple human studies, disruptions of *Nos3* signalling have been identified in ascending aortic walls of BAV patients (Ridnour et al., 2007; Zhang et al., 2011). Moreover, a small patient study reported that polymorphisms in *Nos3* were associated with aortic dissections in patients with thoracic aortic aneurysms (Ekmekcj et al., 2014). These reports suggest that disrupted NO signalling impacts pathologic onset in the human thoracic aorta. Nevertheless, more recently, a large cohort study reported no significant associations between *Nos3* and BAV patients with thoracic aneurysms, suggesting that *Nos3* polymorphisms might even protect against aneurysm development in BAV patients (Gillis et al., 2017). Although the exact role of *Nos3* in thoracic aneurysm, aortic dissection and its relation to BAV is not yet fully understood in humans, these reports support an important role of *Nos3* in maintaining vessel wall integrity.

This study examined the developmental processes involved in aortic aneurysm formation and found dissections in ∼13% of *Nos3^−/−^* mice aged from 1 to 11 months, of which 25% had a BAV. Ultrasound imaging showed that *Nos3^−/−^* mice develop aortic dilations into adulthood, similar to observations in BAV patients. The dissections were a result of disruption in elastin by VSMCs. A reduction in NCC-derived VSMCs that populate the inner aortic media was observed during mid-gestation and late embryonic development of *Nos3^−/−^* mice, supporting a congenital predisposition for development of BAV-associated aortopathy. Single-cell sequencing of embryonic outflow tracts showed significant downregulation of *Eln* and *Fbln5* mRNA in VSMCs of *Nos3^−/−^* mice, which was also confirmed in ascending aortic tissue of adult mice, showing that the embryonic disruptions in elastic lamellae formation persisted into adulthood. Downregulation of *Eln* and *Fbln5* translated into reduced ELN and FBLN5 protein, which primarily affected NCC-derived VSMCs. Disrupted endothelial-mediated NO signalling caused congenital BAV-associated aortic dilation and dissection as a result of inhibited elastic lamellae formation in VSMCs in *Nos3^−/−^* mice.

## MATERIALS AND METHODS

### Animals

BAV-associated aortopathy was studied in aortic tissue of wild-type and *Nos3^−/−^* mice in embryonic and adult stages of development. Mice older than 2 months were considered as adult mice, and experiments were performed using a random distribution of male and female mice. The following mice were used in this study: *Nos3^−/−^* B6.129P2-*Nos3*^*tm1Unc*^/J mice (purchased from Charles River Laboratories, Maastricht, Netherlands), B6.Cg-^*Tg(Wnt1-cre)2Sor*^/J (purchased from The Jackson Laboratory, Bar Harbor, USA; JAX stock 022501), B6.129(Cg)-*Gt(ROSA)26Sor*^*tm4(ACTB-tdTomato,-EGFP)Luo*^/J, (mT/mG) (purchased from The Jackson Laboratory; JAX stock 007576)*. Nos3^−/−^*;*Wnt1Cre;mT/mG* and *Wnt1Cre;mT/mG* were generated using a cross-breeding strategy. All mice were backcrossed to the Black6 background using C57BL/6JLumc mice (purchased from Leiden University Medical Center, Leiden, The Netherlands).

Embryos were acquired using timed breeding protocols. Adult mice were bred overnight and examined the next morning for the presence of a vaginal plug. In cases where a plug was observed, embryonic age would be established at E0.5 at noon of that day. Embryos were isolated through hysterectomy at E12.5 and E17.5 following dissection in phosphate buffer solution pH 7.4. Genomic DNA was isolated from tail biopsies for genotyping by polymerase chain reaction targeted at *Cre* and *Nos3*, as published previously (Peterson et al., 2018). All mice were handled according to the Guide for Care and Use of Laboratory Animals, as published by the NIH, and experiments were in accordance with relevant local, national and international regulations and guidelines.

### Immunostaining and histochemistry

Embryos and adult aortic tissues were fixed in 4% paraformaldehyde (0.1 M, pH 7.4) for 24 h at 4°C and embedded in paraffin. Samples were sectioned serially (5 μm), and mounted on glass slides. Prior to staining, samples were deparaffinized using xylene, followed by a series of graded ethanol steps for rehydration into PBS. In case of immunostaining, slides were subjected to microwave antigen retrieval in citric acid buffer (10 mM citric acid, 0.05% Tween 20, pH 6.0) for 12 min at 97°C. Sections were incubated with primary antibodies against eGFP (Abcam; ab13970), ACTA2 (Sigma-Aldrich; A2547) or FBLN5 (Abcam; ab202977). Primary antibodies were diluted (1:500) in PBS containing Tween-20 and 1% bovine serum albumin (BSA; A8022; Sigma-Aldrich, St Louis, MO, USA) to avoid non-specific binding. Between subsequent incubation steps, all slides were rinsed twice in PBS followed by a single rinse in PBS-Tween-20. Primary antibodies were visualized by incubation with fluorescently labelled secondary antibodies (Thermo Scientific; A-11039), diluted (1:200) in PBS-Tween-20 for 60 min. DAPI (D3571; used at 1:1000; Life Technologies) was used as a nuclear stain, and the slides were mounted with ProLong Gold (Life Technologies). Classical histochemistry was used to examine ECM composition. Mayer's Hematoxylin-Eosin (HE) (KLINIPATH; VWRK4085-9002), Weigert's Resorcin-Fuchsin (RF) (Sigma-Aldrich; 100591) and Sirius Red (Sigma-Aldrich; 365548) staining were performed according to published protocols (Culling, 1974; Junqueira et al., 1979; Cardiff et al., 2014).

### *In vivo* aortic ultrasound measurements

Wild-type (*N*=8; 1 female and 7 males) and *Nos3^−/−^* (*N*=4; 1 female and 3 males) mice were selected at 4 months of age. Mice were randomized, and ultrasound measurements of systolic and diastolic aortic diameters (AoDs and AoDd, respectively) as well as data analysis were executed blinded. Aortic diameters were measured perpendicular to the inner curvature. Mice were anesthetized using isoflurane and monitored for temperature and heart rate during ultrasound measurements. Ultrasound images were collected using a Vevo3100 (FUJIFILM Visual Sonics, Toronto, ON, Canada) and the MX400, 20-46 MHz probe with a centre frequency of 30 MHz. Sagittal ECG-gated kHz visualizations (EKV) were captured for analysis. Data analysis was performed using Vevo LAB 3.2.0 software. Circumferential Green–Lagrange strain was calculated as published previously (Goergen et al., 2010) using the following equation:
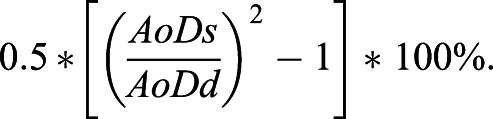


### Three-dimensional reconstructions

Aortic 3D reconstructions of E12.5 *Nos3^−/−^;Wnt1Cre;mTmG* and *Wnt1Cre;mTmG* embryos were made using Amira software 6.3 (Template Graphics Software Inc., Houston, TX, USA). Tissue sections (5 µm) were collected from paraffin-embedded embryos and immunostained using anti-eGFP and DAPI. Slides were scanned using the panoramic 250 flash III slide scanner (3DHISTECH Ltd., Budapest, Hungary), and images of identical scale and exposure were exported using Histech Panoramic Viewer (3DHISTECH Ltd., Budapest, Hungary) then aligned in the Amira software. Relevant cardiac structures were labelled, and surface views were exported to PDF format using the Adobe Acrobat 9.5 software package.

### Extracellular matrix quantification

ECM of elastin and collagen were examined in aortic vessel walls of wild-type (*N*=5) and *Nos3^−/−^* (*N*=6) mice aged E17.5 as well as in wild-type and *Nos3^−/−^* adult mice (2 months; wild type, *N*=6, 2 females and 4 males; *Nos3^−/−^*, *N*=5, 3 females and 2 males). Histological sections were scanned using the panoramic 250 flash III slide scanner (3DHISTECH Ltd., Budapest, Hungary). Elastin and collagen was quantified in aortic vessel wall of the proximal, mid- and distal ascending aorta covering a total length of 250 µm and 500 µm in E17.5 and adult mice, respectively, corresponding to the same anatomical regions within the aorta. This was achieved with the use of a macro designed in Fiji (Schindelin et al., 2012) that allowed for the quantification of volumetric elastin and collagen respective to the vessel wall. The aortic vessel wall was identified manually in histological images (8-bit RGB) of identical resolution and magnification in wild-type and *Nos3^−/−^* mice. Elastin was detected using the following RGB thresholds: hue, min=169, max=227; saturation, min=34, max=255; brightness, min=17, max=185. Collagen was defined using the following RGB thresholds: hue, min=0, max= 255; saturation, min=0, max=255; brightness, min=0, max=199. ECM measurements were presented relative to the volume of aortic vessel wall. Final volumetric calculations were processed in Excel 2016 (Microsoft, Redmond, Washington, USA).

### Survival analysis

Events of spontaneous mortality were recorded in breeding colonies of wild-type and *Nos3^−/−^* mice up to 1 year of age. The number of spontaneous deaths recorded was 103 in wild-type colonies and 133 in *Nos3^−/−^* colonies*.* Mantel–Cox comparison of survival curves was used to examine the temporal distribution of spontaneous death events between the two groups.

### Neural crest lineage analysis

Neural crest lineage analysis was performed similar to analysis in a previous publication (Peterson et al., 2018). Briefly, fluorescence images were collected using a Leica Sp8 confocal microscope (Leica Microsystems, Buffalo Grove, IL, USA). Measurements were performed on aortic vessel walls from transverse sections (5 µm) of *Wnt1Cre;mTmG* (*N*=4) and *Nos3^−/−^;Wnt1Cre;mTmG* (*N*=4) for stages E12.5 and E17.5. Of each embryo, the proximal, mid- and distal ascending aorta were imaged completely covering a total aortic length of 180 µm and 250 µm in E12.5 and E17.5 embryos, respectively, corresponding to the same anatomical regions within the aorta. The proximal ascending aorta marks the border of the sinotubular junction and the tubular ascending aorta. The distal ascending aorta marks the border in which the tubular ascending aorta transitions into the proximal aortic arch prior to the brachiocephalic artery. The mid-ascending aorta is positioned in the middle of the tubular ascending aorta. Image analysis was performed using a macro designed in Fiji (Schindelin et al., 2012). The macro was designed to differentiate the nuclear volume of Wnt1Cre^+^ lineage-derived nuclei from the nuclear volume of all DAPI-positive nuclei within the aortic vessel wall. The regions of the aortic vessel wall were selected manually and DAPI-positive nuclei found within a body of cytoplasmic GFP were measured as lineage-specific nuclei relative to the total volume of DAPI-positive nuclei in the aortic vessel wall. Image thresholds for GFP-positive cytoplasm were set at a pixel intensity of 120 and DAPI-positive thresholds were automatically detected using the ImageJ ‘default’ algorithm. Final volumetric calculations were processed in Excel 2016 (Microsoft, Redmond, WA, USA).

### Cell sorting and single-cell RNA-seq

E12.5 murine *Nos3^−/−^* (*N*=4) and wild-type (*N*=4) embryos were collected in cold PBS, after which the heart was dissected and the cardiac outflow tract was carefully isolated. Cardiac outflow tracts were incubated with 10% trypsin for 7 min at 37°C and resuspended on ice to obtain a single-cell suspension. Cells were washed twice with PBS supplemented with 10% fetal calf serum and transferred over a cell strainer prior to FACS sorting. Dying cells were labelled using DAPI (1:1000) and excluded from further sorting. Single cells were captured using a FACSAria III cell sorter (BD Biosciences) and distributed over 384-well plates containing CEL-Seq2 primer solution and mineral oil, as described previously (Muraro et al., 2016). The 384-well plates were immediately frozen on dry ice and stored at −80°C.

CEL-Seq2 primers and ERCC spike-in RNA (0.02 μl of 1:50,000 dilution) were dispensed with the Mosquito HTS (TTPlabtech). Cell lysis was performed using heat shock incubation of cells for 5 min at 65°C. Reverse transcription and second-strand synthesis reagents were dispensed using the Nanodrop II (GC biotech) to generate barcoded cDNA libraries unique to each cell. The barcoded cDNA libraries in all wells were pooled prior to linear amplification *in vitro*. To generate Illumina sequencing libraries, TruSeq small RNA primers (Illumina) were used for library PCR. Libraries were sequenced using 75-bp paired-end sequencing on an Illumina Nextseq500 platform.

Paired-end reads were mapped to the reference genome GRCm38/mm10 using the Burrows–Wheeler Aligner tool (version 0.7.17) (Li and Durbin, 2010).

The RaceID3 algorithm was used to cluster cells based on K-medoids clustering, as described previously (Herman et al., 2018). RaceID3 analysis was performed using a criteria of mintotal=1000, excluding cells that had lower than 1000 unique transcripts. Further analysis used default parameters. Mitochondrial and ribosomal genes were excluded, because their abundant expression interfered with downstream clustering. Cell clusters were visualized using t-distributed stochastic neighbor embedding (t-SNE), and differential expression of genes between subgroups of cells was calculated using the DESeq2 package in the R platform (Love et al., 2014; Grün and Van Oudenaarden, 2015). The R code and documentation of RaceID3 is available for download at https://github.com/dgrun/RaceID3_StemID2_package (Herman et al., 2018).

### RNA isolation and quantitative real-time PCR

Six-month-old adult mice were euthanized using cervical dislocation, after which the heart and aortic arch were isolated. Aortic samples were carefully dissected from the ascending aorta, minimizing any external tissue contamination. Whole tissue RNA isolation of *Nos3^−/−^* (*N*=5, 2 females and 3 males) and wild-type mice (*N*=5, 2 females and 3 males) was performed using TRIzol reagent (Invitrogen) and gDNA removal was performed using the TURBO DNA-Free kit (Invitrogen), followed by reverse transcription to obtain cDNA using an Iscript cDNA synthesis kit (Bio-Rad) according to manufacturer's protocols.

Quantitative PCR was performed using SYBR Green (Bio-Rad) on a CFX384 Touch Real-Time PCR Detection System (Bio-Rad). The PCR program ran a single cycle of 50°C (10 min) and 95°C (5 min), followed by 40 cycles of 95°C (10 s) and 60°C (1 min). Primers used in qPCR are described in Table S1. qPCR was performed in triplicates, and the average Ct score was quantified relative to the housekeeping genes *Rpl32* and *Gapdh*. Differential gene expression in ascending aortas of wild-type and *Nos3^−/−^* mice was presented as log2 fold change.

### Statistical analysis

Results are presented as mean±s.d. of at least three independent experiments. Statistical analyses were performed using an unpaired two-tailed Student's *t*-test. Significance was assumed when *P*<0.05. Statistical analysis was performed in GraphPad Prism 8.0 for Windows (GraphPad Software, La Jolla California USA).

## Supplementary Material

Supplementary information
