# Supplementary material for: Disturbed nitric oxide signalling gives rise to congenital bicuspid aortic valve and aortopathy
Source: Dis Model Mech. 2020 Sep 28;13(9):dmm044990. doi: 10.1242/dmm.044990 (PMC7541347; doi:10.1242/dmm.044990)
Supplement: Supplementary information [file dmm-13-044990-s1.pdf]

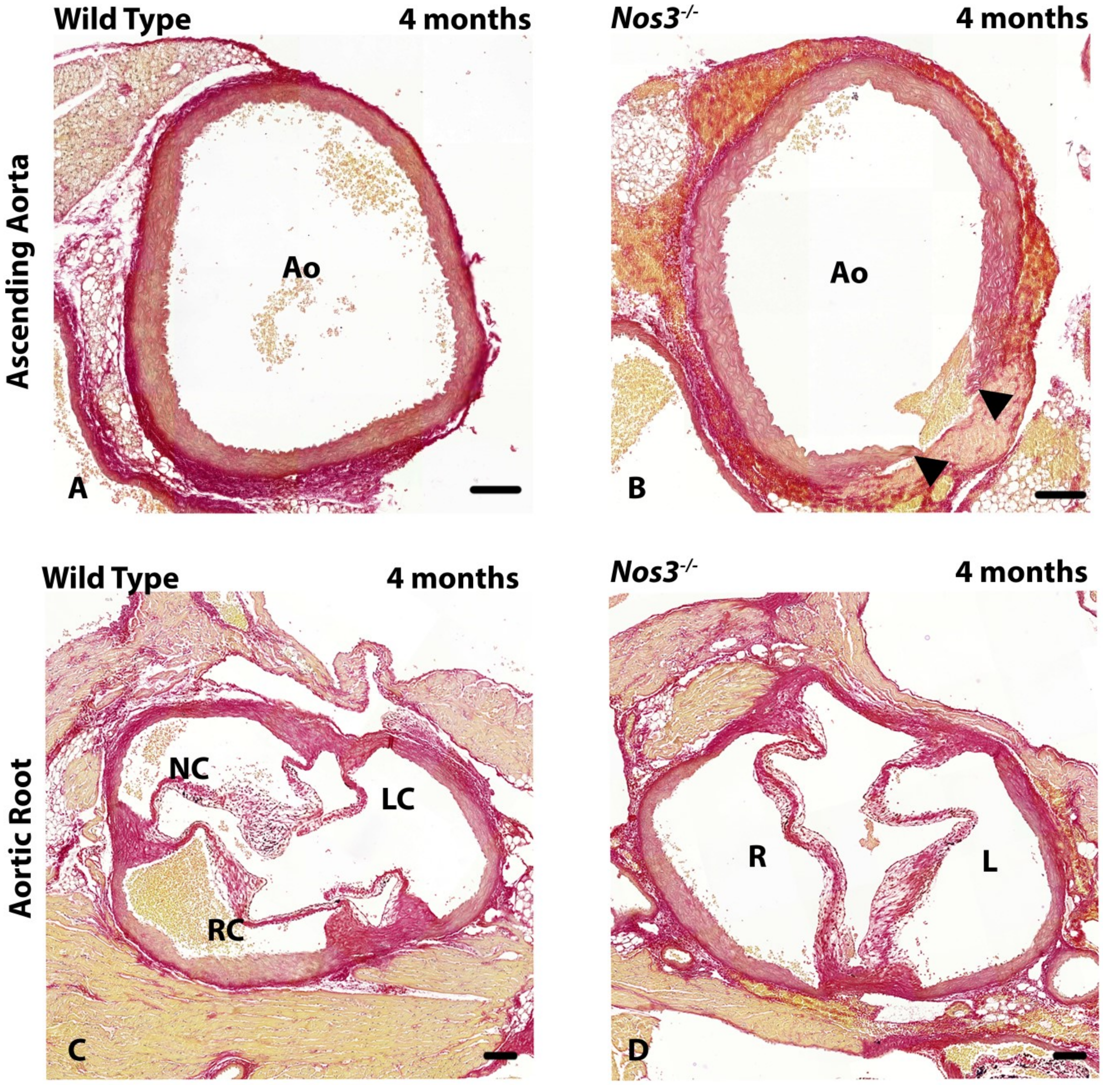

Fig. S1. Aortic dissection in Nos3$^{-/-}$ mice is associated with BAV.

Additional histological staining of wild type and *Nos3*$^{-/-}$ mice presented in figure 1 stained with a combination of collagen (red) and elastin (pink) showing the ascending aorta (A-B) and aortic root (C-D). This case of aortic dissection developed in conjunction with a bicuspid aortic valve (D). Aortic dissection is apparent in the aortic vessel wall of the *Nos3*$^{-/-}$ mouse (arrow heads). Ao: Aorta, NC: Non-coronary leaflet, RC: Right coronary leaflet, LC: Left coronary leaflet, R: Right leaflet, L: Left leaflet, Scale bar: 100 µm

Disease Models & Mechanisms • Supplementary information

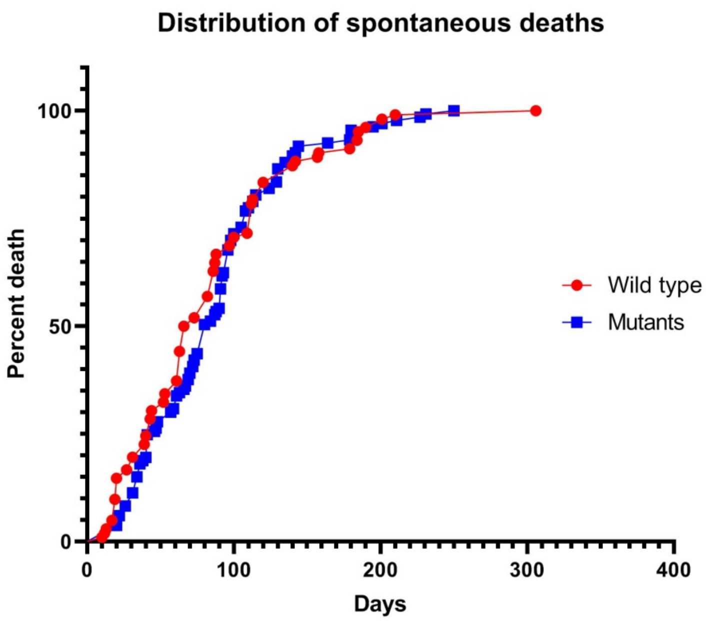

Fig. S2. Temporal distribution of spontaneous death events.

Wild type (n=103) and *Nos3*$^{-/-}$ (n=133) in which spontaneous death was observed were examined for the chronologic distribution of death events. No significant (P >0.05) difference was observed between wild type and mutant populations using Mantel-Cox comparison of survival curves.

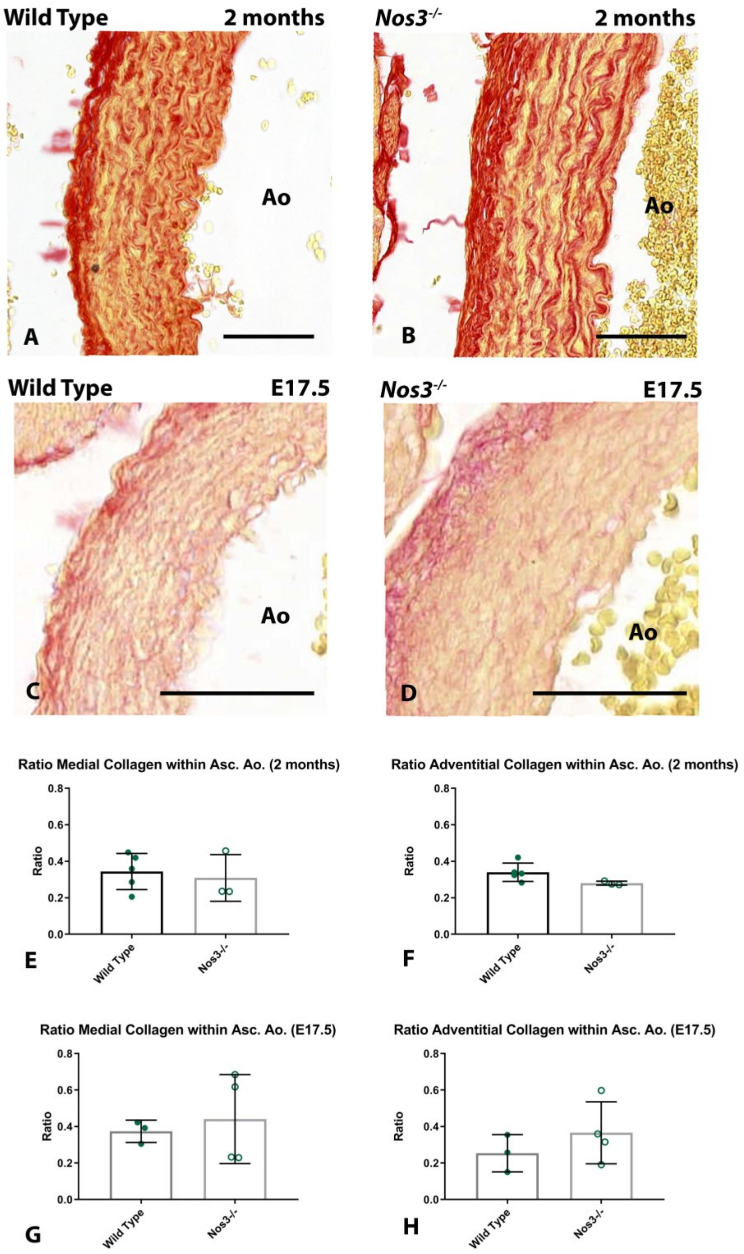

Fig. S3. Collagen deposition is not affected in the ascending aortic wall of Nos3$^{-/-}$ mice. A-B: Transverse sections of the aortic wall of adult (A) wild type and (B) *Nos3*$^{-/-}$ mice stained with Sirius red to show collagen deposition in the media and adventitia of the ascending aorta. C-D: Sirius red staining of the embryonic aortic wall of (C) wild type and (D) *Nos3*$^{-/-}$ mice at stage E17.5. E-H: Volumetric quantification of collagen staining within the medial (E,G) as well as adventitial layers (F,H) of the adult and embryonic ascending aortic wall show no difference ($P > 0.05$) in the deposition of collagen between wild type and *Nos3*$^{-/-}$ mice. Ao: Aorta. Data are mean ± s.d. for $n \geq 3$ mice per group. Scale bar: 50 µm

Disease Models & Mechanisms • Supplementary information

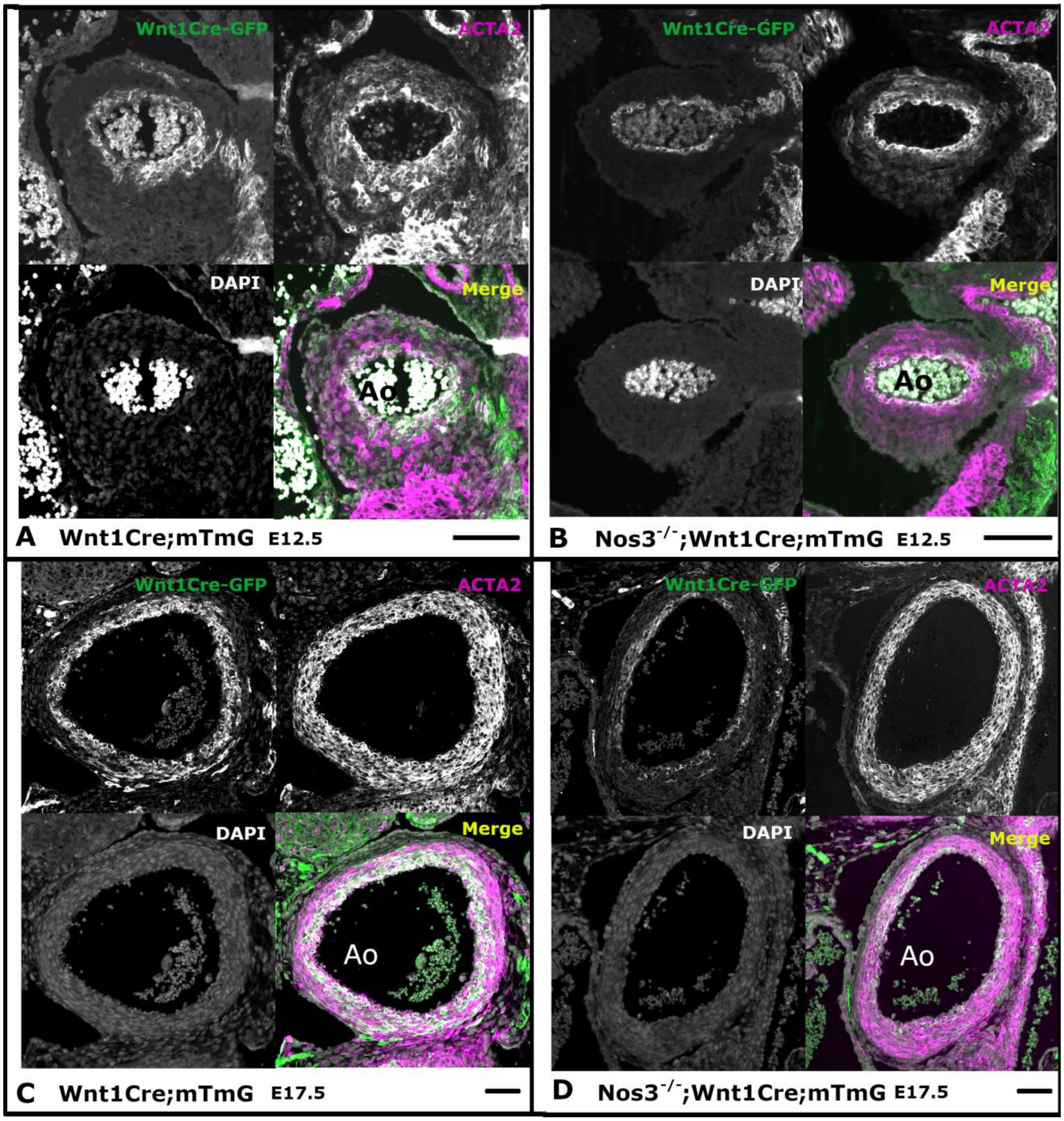

Fig. S4. Neural crest derived smooth muscle cells populate the inner media of tthe ascending aortic vessel wall.

A-B: Transversal sections of the e a ascending aorta of Wnt1Cre;mTmG and *Nos3*$^{-/--}$*;Wnt1Cre;mTmG* embryos at E12E12.5. Neural crest derived vascular smooth muscclle cells (VSMCs) express both Wnt1Cree-GFP (green) and ACTA2 (magenta). C-D: Fluoruorescent images similar to A and B, but shs howing embryos of developmental age E17.5. NNote that expression of ACTA2 is more pronounpronounced in neural crest derived VSMCs than VVSMCs of different origin at E12.5 in both w wild type and *Nos3*$^{-/-}$ embryos. Nuclear staining:ng: DAPI (grey). Scale bars: 50µm. Ao: AorA orta.

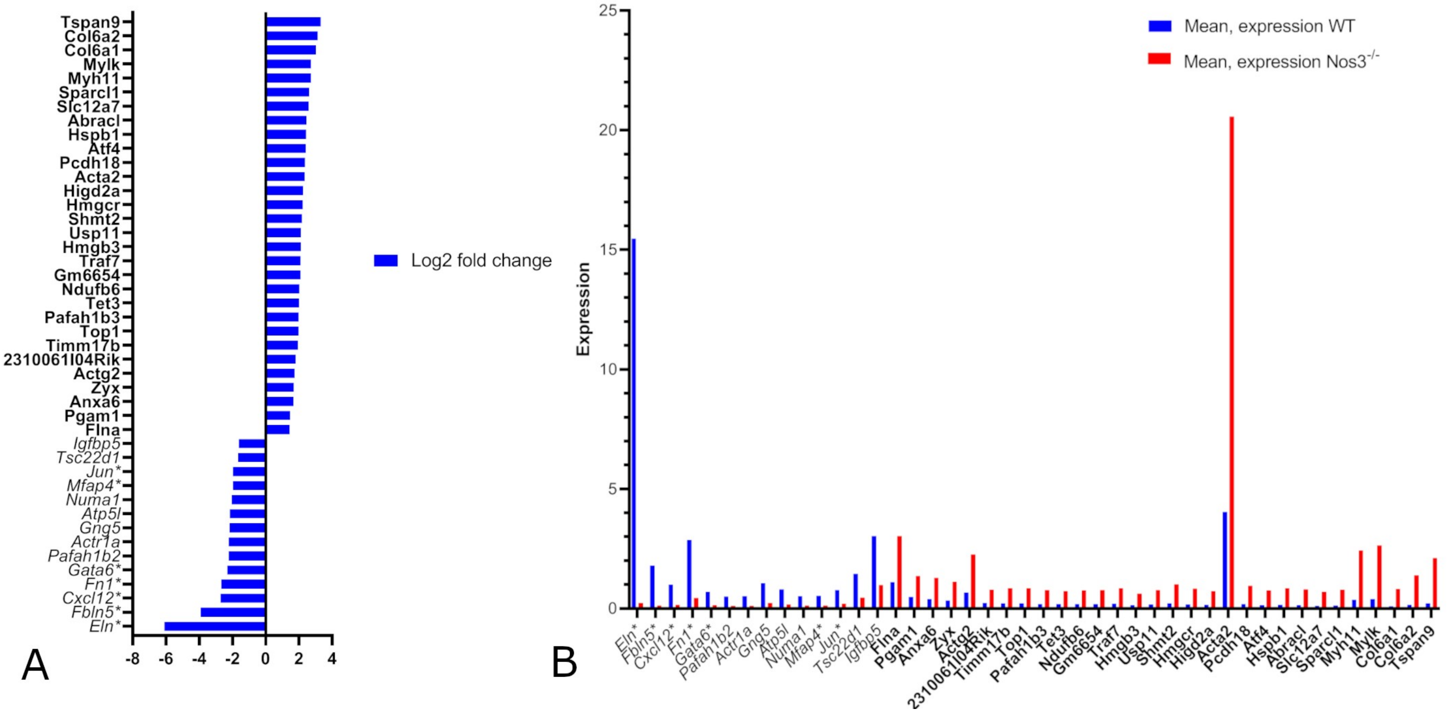

Fig. S5. Differential gene expression profiles among VSMC clusters.

A-B: The differential expression profile of VSMCs affected by the *Nos3* mutation. Astrix indicates downregulated genes associated with aneurysm formation. Bold gene names correspond to upregulated genes.

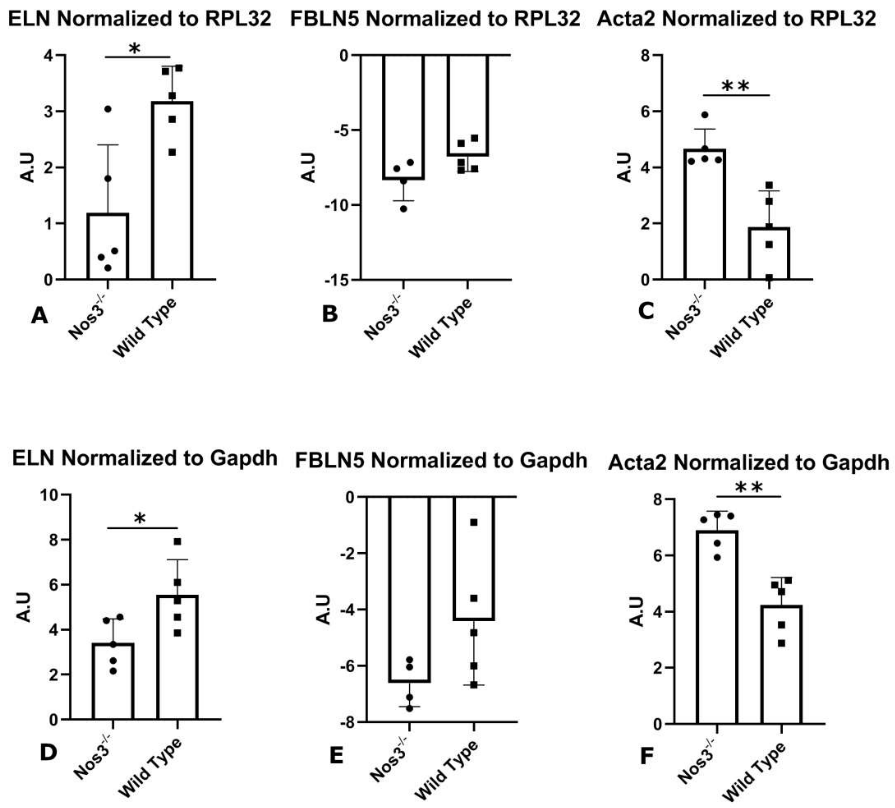

Fig. S6. Extended qPCR evaluatation normalized to Rpl32 and Gapdh.
A-F: qPCR expression results of 6 month old wild type (N=5) and *Nos3*$^{-/-}$ (N=5) mice using *Rpl32* as well as *Gapdh* as reference genes. Statistical analysis were performed using a two-tailed student T-test, * and ** indicate P<0.05 and P<0.01 respectively. A.U: Arbitrary Units. Data are mean ± sd.

Disease Models & Mechanisms • Supplementary information

ELN_FWD	CCC ACC TCT TTG TGT TTC GC
ELN_REV	CCC AAA GAG CAC ACC AAC AAT
FBLN5_FWD	GTG CTT GGG GTT GGT TTT GA
FBLN5_REV	TCA GTT CCC CAT CTT TTG CCA
ACTA2_FWD	GCT ACG AAC TGC CTG ACG G
ACTA2_REV	TAG GTG GTT TCG TGG ATG CC
RPL32_FWD	CAC CAC TCA GAC CGA TAT GTG AAA A
RPL32_REV	TGT TGT CAA TGC CTC TGG GTT T
GAPDH_FWD	TTG ATG GCA ACA ATC TCC AC
GAPDH_REV	CGT CCC GTA GAC AAA ATG GT

Table S1. Primers used for qPCR
